# Estimation of True Serum Thyroglobulin Concentration Using Simultaneous Measurement of Serum Antithyroglobulin Antibody

**DOI:** 10.1155/2013/210639

**Published:** 2013-03-31

**Authors:** Byeong-Cheol Ahn, Won Kee Lee, Shin Young Jeong, Sang-Woo Lee, Jaetae Lee

**Affiliations:** ^1^Department of Nuclear Medicine, Kyungpook National University School of Medicine/Hospital, 50 Samduk-dong 2-ga, Chung Gu, Daegu 700-721, Republic of Korea; ^2^Department of Preventive Medicine, Kyungpook National University School of Medicine/Hospital, 50 Samduk-dong 2-ga, Chung Gu, Daegu 700-721, Republic of Korea

## Abstract

We investigated the analytical interference of antithyroglobulin antibody (TgAb) to thyroglobulin (Tg) measurement and tried to convert measured Tg concentration to true Tg concentration using a mathematical equation which includes a concentration of TgAb. *Methods.* Tg was measured by immunoradiometric assay and TgAb by radioimmunoassy. Experimental samples were produced by mixing Tg and TgAb standard solutions or mixing patients' serum with high Tg or high TgAb. Mathematical equations for prediction of expected Tg concentration with measured Tg and TgAb concentrations were deduced. The Tg concentration calculated using the equations was compared with the expected Tg concentration. *Results.* Measured Tg concentrations of samples having high TgAb were significantly lower than their expected Tg concentration. Magnitude of TgAb interference with the Tg assay showed a positive correlation with concentration of TgAb. Mathematical equations for estimation of expected Tg concentration using measured Tg and TgAb concentrations were successfully deduced and the calculated Tg concentration showed excellent correlation with expected Tg concentration. *Conclusions.* A mathematic equation for estimation of true Tg concentration using measured Tg and TgAb concentration was deduced. Tg concentration calculated by use of the equation might be more valuable than measured Tg concentration in patients with differentiated thyroid cancer.

## 1. Introduction

Thyroglobulin (Tg), a glycoprotein synthesized in normal or malignant thyroid follicular cells, is an important marker for residual or recurrent differentiated thyroid cancer. Undetectable Tg is one of the criteria to establish the absence of a persistent tumor or recurrence in patients with differentiated thyroid cancer who have undergone total thyroidectomy and remnant ablation with radioiodine [1, 2]. Tg is the most sensitive marker for detecting recurrence of differentiated thyroid cancer; however, the presence of antithyroglobulin antibody (TgAb) interferes with measurement of Tg; therefore, development of Tg assays with limited or no interference by TgAb and development of methods for clearing of TgAb prior to measurement of Tg are warranted [1, 3, 4]. Until now, no TgAb-proof Tg assay (Tg assay without influence of TgAb) has been made available, and the presence of TgAb causes the concentration of measured Tg to be lower than that of the true concentration [4–6].

 In patients with differentiated thyroid cancer who underwent curative treatment with total thyroidectomy followed by high-dose radioiodine ablation, the cut off value of Tg for performance of imaging studies for detection of persistent disease or recurrence is variable, according to the status of TSH and the concentration of measured TgAb [1]. Despite the lack of an international consensus regarding the appropriate Tg cut off value for residual or recurrent disease [7], almost all institutions or physicians have their own cut off value for predicting persistent or recurrent disease according to TSH status (stimulated or not stimulated). Another factor to consider in interpretation of measured Tg value is the presence or absence of TgAb, the strongest serologic factor interfering in accuracy of available Tg assays [3, 8]. Measurement of TSH-stimulated Tg can result in failure to identify significant persistent or recurrent tumors in patients with TgAb. Influential magnitude of TgAb on measurement of Tg is known to show correlation with the concentration of measured TgAb [4]. In addition, it has been also known that Tg radioimmunoassay is less prone to the influence than other immunometric assays. Recently, Locsei et al. reported that decrease of measured Tg concentration by adding sheep TgAb from the electrochemiluninometric Tg assay and the magnitude of the influence was significant even in the reference range [9].

 In this study, the authors assessed the influence of TgAb on measurement of Tg and developed a mathematical equation for estimation of true Tg concentration under various concentrations of TgAb using data from experiments that employed both standard solutions of Tg and TgAb measurement kits and patients' serum having high Tg or high TgAb.

## 2. Materials and Methods

### 2.1. Tg Measurements

Tg was measured by immunoradiometric assay (IRMA) using a commercial reagent set (Dynotest Tg-plus; Brahms Diagnostica, Berlin, Germany, detection limit; 0.08 ng/mL, measuring range; up to 250 ng/mL) according to the manufacturer's recommendations. The method described by the manufacturer is as follows. Standard solution or experimental serum (100 *μ*L) is pipetted into test tubes coated with polyclonal TgAb. The tubes are then incubated for 18 hours at room temperature, and washed twice with 2 mL of washing solution. The tubes are turned upside down on blotting paper for at least 10 minutes. The tubes are again turned right side up, followed by addition of 200 *μ*L of ^125^I-labeled monoclonal TgAb. The tubes are incubated for 2-3 hours at room temperature with shaking (300–400 rpm), followed by washing twice with 2 mL of washing solution. The tubes are then turned upside down again on blotting paper for at least 10 minutes. Radioactivity of each tube is then measured. Concentration of Tg is obtained using a standard curve derived using the standard solutions.

### 2.2. TgAb Measurements

TgAb was measured by radioimmunoassay (RIA) using a commercial reagent set (Dynotest anti-Tgn; Brahms Diagnostica, Berlin, Germany, detection limit; 5.5 U/mL, measuring range; up to ~2000 U/mL) according to the manufacturer's recommendations. The method described by the manufacturer is as follows: standard solution or test serum (20 *μ*L) is pipetted into test tubes coated with polyclonal anti-TgAb, followed by addition of 200 *μ*L of ^125^I-labeled Tg to the tubes. The tubes are incubated for 2 hours at room temperature with shaking (300–400 rpm), followed by washing three times with 2 mL of washing solution. The tubes are then turned upside down on blotting paper for at least 10 minutes. Radioactivity of each tube is then measured. Concentration of TgAb is obtained using a standard curve derived using standard solutions.

### 2.3. Preparation of Experimental Samples

Several concentrations of Tg standard solutions (4.0, 20.0, 100.0, and 250.0 ng/mL, Dynotest Tg-plus) and several concentrations of TgAb standard solutions (20.0, 60.0, 200.0, 600.0, and 2000.0 U/mL, Dynotest anti-Tgn) were prepared. In order to generate experimental samples containing various concentrations of Tg and TgAb, equal volumes of standard solutions were mixed (Table 1). Serum samples containing various concentrations of Tg (9.3, 37.6, 221.9, and 492.0 ng/mL) with a low level of TgAb (<20 U/mL) and serum samples containing various concentrations of TgAb (7.0, 10.4, 37.5, 1286.0, and 1860.0 U/mL) without Tg (<0.1 ng/mL) were collected. All the serum samples were obtained from patients with differentiated thyroid cancer. In order to generate experimental samples with various concentrations of Tg and TgAb, equal volumes of serum samples were also mixed (Table 2). In order to test reproducibility of measured Tg concentration for the experimental samples, triple samples were prepared for each concentration of every experimental sample produced using standard solutions or patients' serum.

### 2.4. Statistics and Deduction of Equations for Prediction of True Tg

Reproducibility of Tg measurement was tested. Influence of TgAb on measurement of Tg was analyzed and equations predicting expected (true) Tg concentration with measured Tg and TgAb concentration were deduced using the SAS program (version 9.22, SAS Institute Inc., Cary, NC, USA). *P* < 0.05 was considered significant.

## 3. Results

### 3.1. Reproducibility of Tg Measurement

Reproducibility of Tg measurement performed on triplicate samples of each concentration of experimental samples produced using either standard solution or patients' serum was found to be excellent. Coefficient of variation for experimental samples produced using standard solutions was 4.21 ± 3.51% (0 ~ 14.82) (intraclass correlation coefficient = 0.998). Coefficient of variation for experimental samples produced using serum from patients was 2.83 ± 2.23% (0.87 ~ 11.21) (intraclass correlation coefficient = 0.999). 

### 3.2. Influence of TgAb on Measurement of Tg Using Samples Produced from Standard Solutions

**Figure 1:**
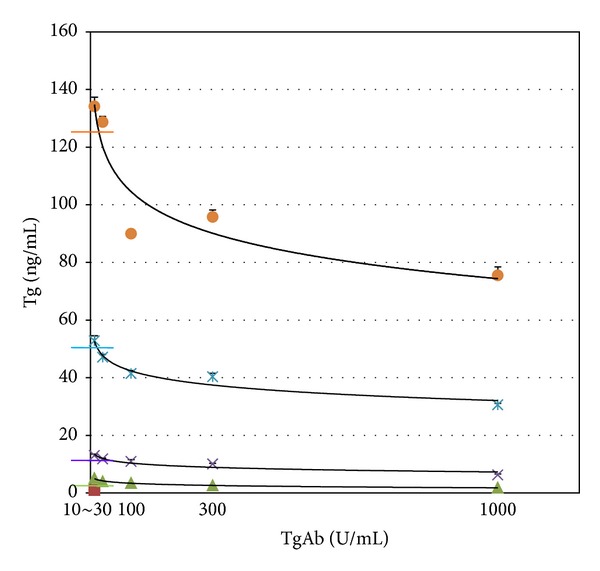
Measured Tg concentration showed a proportional decline according to increase of TgAb concentration in every sample produced using standard solutions. Transverse color bars represent expected Tg concentrations of each sample.

Measured Tg concentration showed a proportional decline with increase of TgAb concentration in every sample produced using standard solutions. Measured Tg concentrations in samples having the lowest concentration (10 U/mL) of TgAb were higher than the expected Tg concentrations of the samples. However, measured Tg concentrations in samples having high TgAb were lower than expected Tg concentrations (Table 3, Figure 1).

### 3.3. Influence of TgAb on Measurement of Tg Using Samples Produced from Patients' Serum

**Figure 2:**
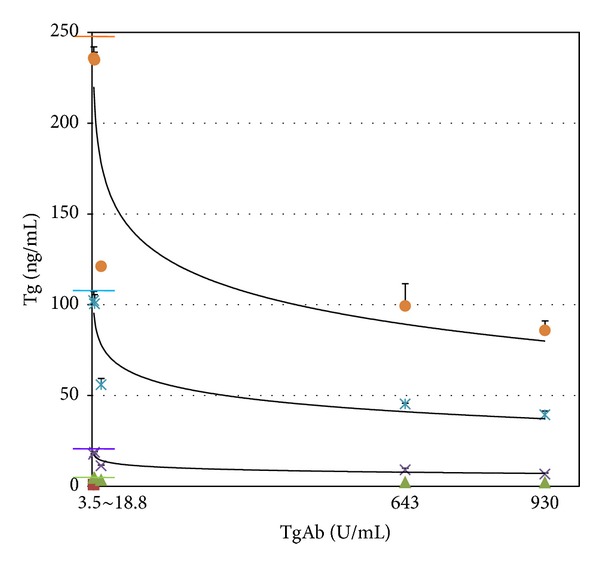
Measured Tg concentration in each sample produced using patients' serum showed a proportional decline according to an increase of TgAb concentration. Transverse color bars represent expected Tg concentrations of each sample.

Measured Tg concentration showed a decline with increase of TgAb concentration in every sample produced using patients serum. Measured Tg concentrations for all samples were found to be lower than expected Tg concentrations (Table 4, Figure 2). 

### 3.4. Equations for Prediction of Expected Tg Concentration

Data obtained with standard solution was used in deduction of an equation for prediction of expected Tg concentrations with measured Tg and TgAb concentrations using the SAS program.

(1) Calculated  Tg (ng/mL)  =−1.553+0.592{measured  Tg (ng/mL)            ×log⁡⁡TgAb (U/mL)}.

**Figure 3:**
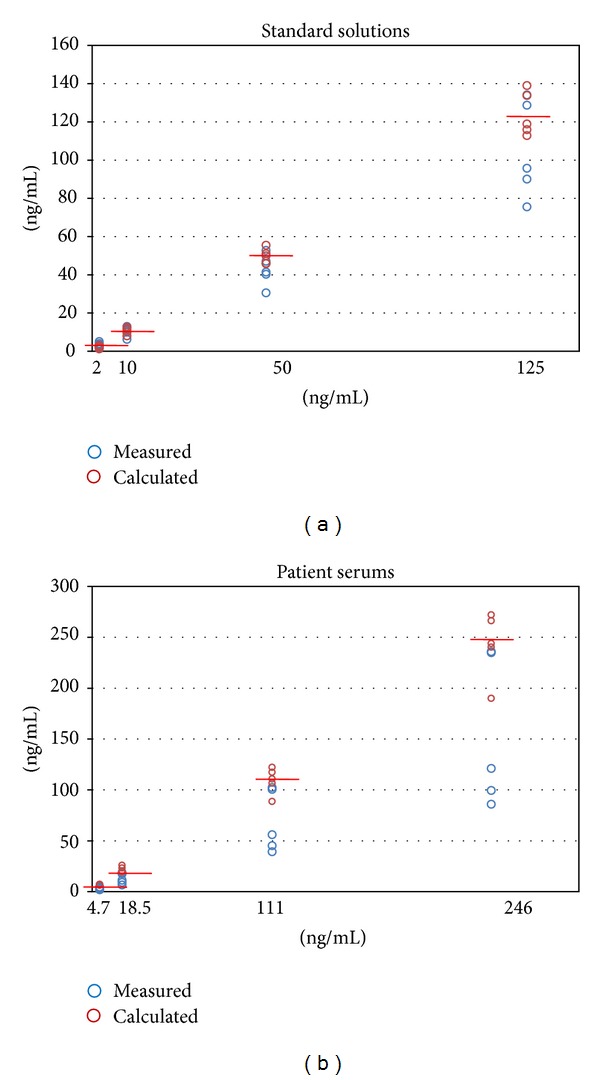
For each concentration of TgAb, the calculated Tg concentrations were more similar to expected Tg concentrations than measured Tg concentrations in samples from both standard solution and patients' serum.

**Figure 4:**
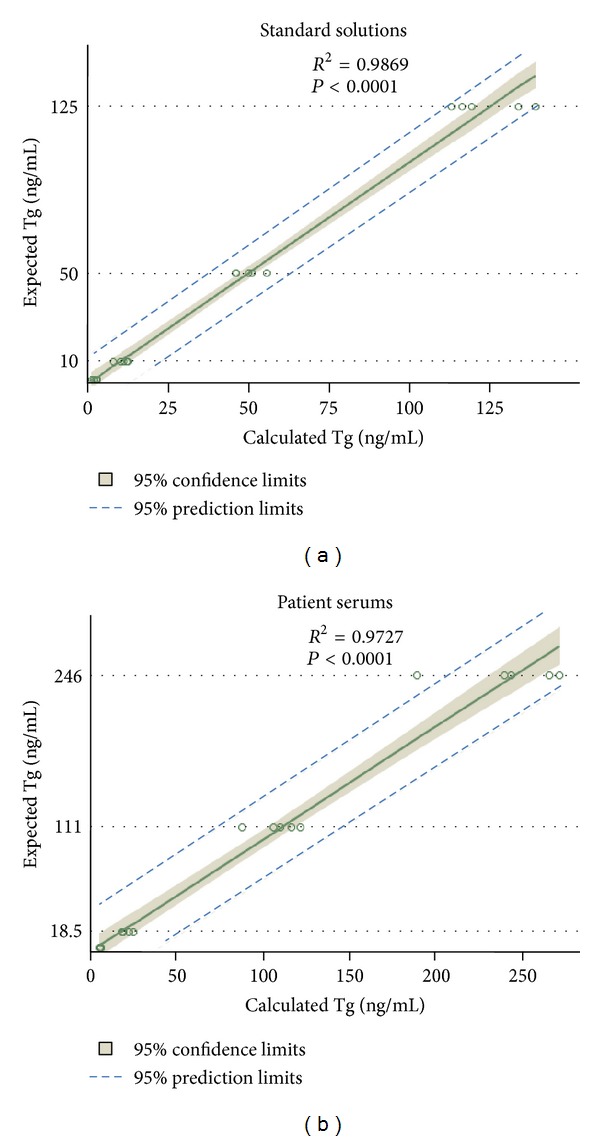
Correlations between the calculated Tg and expected Tg concentrations were found to be excellent in samples produced from both standard solution and patients' serum.

Calculated Tg concentrations were found to be more similar to expected Tg concentrations than measured Tg concentrations, and correlation between calculated Tg and expected Tg concentrations was found to be excellent (*r* ^2^ = 0.9869, *P* < 0.0001) (Figures 3 and 4).

In addition, data obtained with patient serum was used in deduction of an equation for prediction of expected Tg concentrations with measured Tg and TgAb concentrations using the SAS program.

(2) Calculated  Tg (ng/mL)  =1.677+0.634  measured  Tg (ng/mL)   + 0.313  Measured  Tg (ng/mL)   ×log⁡⁡TgAb (U/mL).

Calculated Tg concentrations were found to be more similar to expected Tg concentrations than measured Tg concentrations, and correlation between calculated Tg and expected Tg concentrations was found to be excellent (*r* ^2^ = 0.9727, *P* < 0.0001) (Figures 3 and 4).

## 4. Discussion

Although the majority of patients with differentiated thyroid cancer are apparently rendered disease-free by initial treatment, approximately 15% experience persistent or recurrent cancer [10, 11]. Persistent disease or recurrence can be predicted by measurement of serum Tg, a sensitive and specific tumor marker for detection of differentiated thyroid cancer. Currently, the cut off value of 2 ng/mL under endogenous TSH or recombinant human TSH-stimulation is considered to represent significant risk [1, 10]. However, cut off values from 2 to 30 ng/mL, for example, 10 ng/mL, have also been applied in other clinical studies [7, 12].

Detectable TgAb is reported to be associated with persistence of an antigenic stimulus, and up to 40% of patients with differentiated thyroid cancer are positive for TgAb [13–15]. Some reports have suggested that persistence of TgAb positivity might suggest persistent or recurrent disease in some cases of differentiated thyroid cancer; however, other studies have reported no correlation between TgAb level and disease persistence [16, 17]. Therefore, the most important clinical issue with regard to high serum TgAb concentration is interference of the result of Tg assays with recurrence work up in patients with differentiated thyroid cancer [8, 14, 17, 18]. 

Endogenous TgAb is known to interfere with measurement of Tg in a method-dependent manner; therefore, prediction of Tg under a certain TgAb condition can be method-dependent as well [5]. Data found in the literature indicated that in the presence of TgAb, values of Tg determined by immunoradiometric assay are usually lower than real values, even if the concentrations of TgAb are very low [5, 9, 19]. In previous reports, we observed an erroneously low measured Tg value according to the presence of TgAb, and the degree showed positive correlation with concentration of TgAb [3, 4]. In the current study, influence of TgAb on the measurement of Tg was tested with experimental samples made by Tg and TgAb standard solutions or patients' serum. Two different equations which predict true Tg value were successfully deduced with the result from the tests, and the equation from the patients serum would be more appropriate for clinical application. According to findings from the current study, true Tg values in high concentrations of TgAb are more than twice the measured values. Serum with a true Tg value of 4.7 ng/mL can be measured as 2 ng/mL in samples containing a TgAb concentration of 1860 U/mL. It can be assumed that measured Tg value for a patient with a Tg of 4.7 ng/mL and a TgAb greater than 1860 U/mL might be a Tg of less than 2.0 ng/mL using the Tg assay. As a result, when applying a Tg cut off value of 2.0 ng/mL, the patient can be misclassified as low risk for recurrent or persistent disease. Serum with a true Tg value of 18.5 ng/mL can be measured as 9.1 ng/mL in samples containing a TgAb concentration of 1286 U/mL. It can also be assumed that measured Tg value for a patient with a Tg of 18.5 ng/mL and TgAb greater than 1286 U/mL might be a Tg of less than 10 ng/mL using the Tg assay. As a result, when applying a Tg cut off value of 10 ng/mL, the patient can be misclassified as low risk for recurrent or persistent disease. 

Higher incidence of positive TgAb in patients with differentiated thyroid cancer, compared with the general population, has been reported. In addition, some patients have a high concentration of TgAb [14, 18]. Considering the results of the current study, some patients with a borderline Tg value can be misclassified into a low risk group and therefore would not undergo further diagnostic evaluation to detect recurrence or persistent disease. Management of disease can be delayed and prognosis of patients might be worse than that for patients diagnosed earlier with recurrence or persistent disease.

Consideration of TgAb when deciding on the clinical significance of Tg value has been basically by the presence or absence of TgAb only [1, 20]. It had been generally regarded that TgAb titer measured is below a clinical threshold will not be a significant influence on the Tg outcome; however, recent studies demonstrated that TgAb below the cut off can interfere the Tg outcome [19, 21]. Recently, Locsei et al. also reported that the measured Tg value of patients serum can be influenced by mixing sheep TgAb in the reference range of TgAb concentration and deduced an equation estimating true Tg concentration using TgAb concentration in the same sample [9]. They proved the general concept of TgAb influence on the Tg measurement; however, their equation cannot be generally applied to clinical practice due to difference between sheep TgAb and human TgAb. Verification of the TgAb influence using human TgAb, not sheep TgAb, is needed for that purpose.

In the current study, we used human TgAb from patients' serum for assessment of the influence of Tg to the Tg assay, and verified the same significant influence of human TgAb in reference range to the Tg assay. Results of this study demonstrated that concentration of human TgAb in the reference range also can result in a significantly lower measured Tg value, and a high concentration of TgAb can result in the measured Tg value even lower; therefore, development of methods for use by clinicians in consideration of concomitant low or high concentration of TgAb for determination of the clinical significance of measured Tg values is a pressing issue. In contrary to experiment employing patients' serums, low concentration of TgAb incurred an overestimation of Tg in the experiment employing the standard solutions and elucidation of the cause was not performed in the current study.

Magnitude of the influence is known to not only depend on the class of assay methods, but also the type of Tg epitope recognized by patient's TgAb [16, 18, 22]. Therefore, development of an equation that can be applied to all assay methods and all patients might not be possible. In this study, there was a large deviation of many of the actual points from the curve fits on Figures 1 and 2, suggesting that the back calculation of the true Tg value according to the equation might give quite erroneous results in some patients. The deviation probably originates from the interpatient variability of influence magnitude related to heterogeneity of patients' TgAb. However, results of the current study demonstrated that the Tg value calculated by the equation is generally close to the true Tg value than the measured Tg value. Based on the results, the corrected Tg value by the equation might be more valuable than measured Tg value for predicting the presence or recurrence of a cancerous lesion in patients with differentiated thyroid cancer. However, in fact, clinical validation studies are needed for allowing physician to implement the approach in clinical laboratory practice.

This study has limitations. First, despite efforts to standardize thyroglobulin analytes across assay platforms, differences between platforms persist and can be related to genetic polymorphisms that introduce changes in protein primary structure, glycosylation pathways which could lead to variable protein processing, modification, or cross-linking [6, 8]. Result of TgAb assays was also known to be discordant by their epitope pattern, especially in patients without thyroiditis [23]. The equation would differ according to the assay platforms used for measurement of Tg and TgAb and it cannot be generalized. Therefore, institution's own equation has to be developed by the specific combination of Tg and TgAb assays used. Second, we did not evaluate the influence of Tg on measurement of TgAb. For estimation of true Tg value using measured Tg and TgAb values, the true TgAb value should be plugged into the equation. The influence of Tg on TgAb assay must also be considered [18]. Third, expected Tg and TgAb values might be inaccurate in samples produced using patients serum owing to presence of Tg in serum for TgAb and presence of TgAb in sera for Tg, albeit they are very low in titer. Fourth, in this study, we used only four concentrations of Tg and five concentrations of TgAb. Therefore, the equation formula for estimating true Tg concentration is not the most accurate one, and further studies are needed in order to develop the most accurate equation for estimation of true Tg concentration using measured Tg and TgAb concentrations.

 In conclusion, findings from this study demonstrate a mathematic equation for prediction of true Tg concentration using measured Tg and TgAb concentrations. The true Tg concentration calculated by the equation might be more valuable than measured Tg value for predicting the presence of residual or recurrent cancerous lesions in patients with differentiated thyroid cancer.

## Figures and Tables

**Table 1:** Expected Tg and TgAb concentrations of twenty experimental samples produced with standard solutions of Tg and TgAb. Values are expressed as Tg (ng/mL)-TgAb (U/mL).

2.0-10.0	2.0-30.0	2.0-100.0	2.0-300.0	2.0-1000.0
10.0-10.0	10.0-30.0	10.0-100.0	10.0-300.0	10.0-1000.0
50.0-10.0	50.0-30.0	50.0-100.0	50.0-300.0	50.0-1000.0
125.0-10.0	125.0-30.0	125.0-100.0	125.0-300.0	125.0-1000.0

**Table 2:** Expected Tg and TgAb concentrations of twenty experimental samples produced with patient serums. Values are expressed as Tg (ng/mL)-TgAb (U/mL).

4.7-3.5	4.7-5.2	4.7-18.8	4.7-643.0	4.7-930.0
18.5-3.5	18.5-5.2	18.5-18.8	18.5-643.0	18.5-930.0
111.0-3.5	111.0-5.2	111.0-18.8	111.0-643.0	111.0-930.0
246.0-3.5	246.0-5.2	246.0-18.8	246.0-643.0	246.0-930.0

**Table 3:** Decline of measured Tg value by TgAb in samples produced using Tg and TgAb standard solutions.

Expected Tg concentration (ng/mL)	Measured Tg concentration (ng/mL)				
	under various TgAb concentrations				
	10.0 U/mL	30.0 U/mL	100.0 U/mL	300.0 U/mL	1000.0 U/mL
2.0	5.1 ± 0.1	4.0 ± 0.2	3.4 ± 0.1	2.6 ± 0.1	1.8 ± 0.1
10.0	13.0 ± 0.5	11.8 ± 0.4	10.9 ± 0.7	10.0 ± 0.3	6.2 ± 0.3
50.0	52.8 ± 1.8	47.1 ± 1.0	41.4 ± 1.1	40.3 ± 1.2	30.6 ± 0.5
125.0	134.1 ± 3.2	128.7 ± 2.0	90.0 ± 1.0	95.7 ± 2.5	75.5 ± 3.0

Values are expressed as mean ± SD.

**Table 4:** Decline of measured Tg value by mixed TgAb in samples produced using patients' serum.

Expected Tg concentration (ng/mL)	Measured Tg concentration (ng/mL)				
	under various TgAb concentrations				
	3.5 U/mL	5.2 U/mL	18.8 U/mL	643.0 U/mL	930.0 U/mL
4.7	4.5 ± 0.2	4.5 ± 0.2	3.4 ± 0.6	2.1 ± 0.1	2.0 ± 0.2
18.5	17.6 ± 0.3	18.7 ± 0.4	11.3 ± 0.4	9.1 ± 0.9	6.7 ± 0.6
111.0	102.3 ± 5.1	100.5 ± 5.1	56.0 ± 3.4	45.3 ± 0.4	39.3 ± 2.2
246.0	235.8 ± 6.2	234.8 ± 4.2	121.1 ± 1.9	99.4 ± 12.2	85.9 ± 5.2

Values are expressed as mean ± SD.
